# Poly[[aqua­(μ_7_-biphenyl-3,3′,4,4′-tetra­carboxyl­ato)(1,10-phenanthroline)dicobalt(II)] monohydrate]

**DOI:** 10.1107/S1600536811028224

**Published:** 2011-07-23

**Authors:** Hailiang Yin, Fengjuan Yin, Yukun Lu

**Affiliations:** a College of Chemistry and Chemical Engineering, China University of Petroleum, Qingdao 255666, People’s Republic of China; b Environmental Protection Bureau of Dongying, Dongying 257091, People’s Republic of China

## Abstract

In the title compound, {[Co_2_(C_16_H_6_O_8_)(C_12_H_8_N_2_)(H_2_O)_2_]·H_2_O}_*n*_, one Co^II^ ion has a {CoN_2_O_4_} distorted octa­hedral environment defined by two N atoms of one 1,10-phenanthroline (phen) ligand, three O atoms of the carboxyl­ate groups of three biphenyl-3,3′,4,4′-tetra­carboxyl­ate (BPTC) ligands, one of which is bidentate, and one O atom from one coordinated water mol­ecule. The other Co^II^ atom is surrounded by six O atoms from four different BPTC ligands and one coordinated water mol­ecule. Each BPTC ligand forms eight coordination bonds with seven Co^II^ atoms, leading to a layer structure along the *ac* plane. Uncoordinated water mol­ecules occupy the space between the layers, and inter­act *via* inter­layer O—H⋯O hydrogen bonds along the *b* axis, generating a three-dimensional supra­molecular network.

## Related literature

For applications of compounds with metal-organic framework structures (MOFs), see: Rowsell & Yaghi (2005 ▶). For related structures, see: Zhu *et al.* (2008 ▶); Konar *et al.* (2004 ▶).
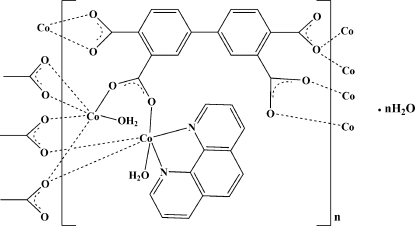


## Experimental

### 

#### Crystal data

[Co_2_(C_16_H_6_O_8_)(C_12_H_8_N_2_)(H_2_O)_2_]·H_2_O
*M* *_r_* = 678.32
Triclinic, 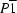

*a* = 9.793 (3) Å
*b* = 10.885 (3) Å
*c* = 12.453 (3) Å
α = 97.567 (4)°
β = 102.608 (4)°
γ = 95.653 (4)°
*V* = 1273.1 (6) Å^3^
*Z* = 2
Mo *K*α radiation
μ = 1.38 mm^−1^
*T* = 298 K
0.40 × 0.17 × 0.16 mm

#### Data collection

Bruker SMART CCD area-detector diffractometer
Absorption correction: multi-scan (*SADABS*; Bruker, 2000 ▶) *T*_min_ = 0.609, *T*_max_ = 0.810
6716 measured reflections
4641 independent reflections
3194 reflections with *I* > 2σ(*I*)
*R*_int_ = 0.034

#### Refinement

*R*[*F* ^2^ > 2σ(*F* ^2^)] = 0.052
*wR*(*F* ^2^) = 0.113
*S* = 0.98
4641 reflections
388 parameters
H-atom parameters constrained
Δρ_max_ = 0.46 e Å^−3^
Δρ_min_ = −0.50 e Å^−3^

### 

Data collection: *SMART* (Bruker, 2000 ▶); cell refinement: *SAINT* (Bruker, 2000 ▶); data reduction: *SAINT*; program(s) used to solve structure: *SHELXS97* (Sheldrick, 2008 ▶); program(s) used to refine structure: *SHELXL97* (Sheldrick, 2008 ▶); molecular graphics: *SHELXTL* (Sheldrick, 2008 ▶); software used to prepare material for publication: *SHELXTL*.

## Supplementary Material

Crystal structure: contains datablock(s) I, global. DOI: 10.1107/S1600536811028224/bg2411sup1.cif

Structure factors: contains datablock(s) I. DOI: 10.1107/S1600536811028224/bg2411Isup2.hkl

Additional supplementary materials:  crystallographic information; 3D view; checkCIF report

## Figures and Tables

**Table 1:** Hydrogen-bond geometry (Å, °)

*D*—H⋯*A*	*D*—H	H⋯*A*	*D*⋯*A*	*D*—H⋯*A*
O11—H11*B*⋯O7^i^	0.85	2.04	2.886 (6)	173
O11—H11*A*⋯O7^ii^	0.85	2.15	2.931 (5)	152
O10—H10*B*⋯O7^iii^	0.85	2.20	2.668 (4)	115
O10—H10*A*⋯O2^iv^	0.85	1.95	2.756 (4)	159
O9—H9*A*⋯O3	0.85	2.09	2.661 (4)	124

Symmetry codes: (i) 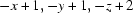; (ii) 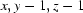; (iii) 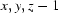; (iv) 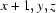.

**Fig. 1.**
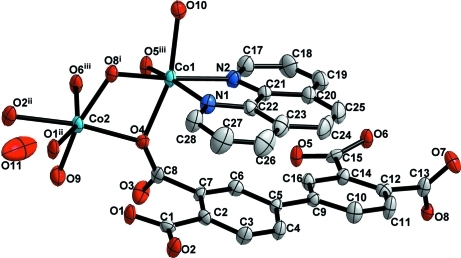
: View of (I), showing 30% displacement ellipsoids. Symmetry codes: (i) x, y, z - 2; (ii) -x, 1 - y, 1 - z; (iii) 1 - x, 1 - y, 2 - z.

**Fig. 2.**
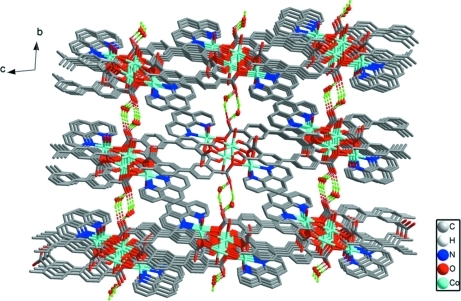
: Crystal packing of (I) as viewed down the crystallographic a axis.

## supplementary crystallographic information

### Comment

The assembly of coordination architectures has attracted much attention in recent years due to their potential applications in separation, sorption, hydrogen storage, and catalysis, as well as due to their intriguing topologies such as molecular ladders, grids, rings, boxes, honeycombs, and diamondoids (Rowsell & Yaghi, 2005). Coordination polymers containing biphenylpolycarboxylate and 1,10-phenanthroline as ligands have played a important role in the area of modern coordination chemistry.  A few coordination polymers dealing with 3,3',4,4'-biphenyltetracarboxylate (H_4_BPTC) and 1,10-phenanthroline (phen) have been reported (Zhu *et al.*,  2008). Herein, we report a new cobalt coordination polymer, {[Co_2_(C_16_H_6_O_8_)(C_12_H_8_N_2_)(H_2_O)_2_]H_2_O}_n_, resulting from reaction of Co^2^^+^ cations, phen and H_4_BPTC under hydrothermal conditions.

As shown in Fig. 1, the asymmetric unit consists of two crystallographically independent  Co^2+^ ions, one fully deprotonated BPTC^4-^ anion, a chelating phen ligand, two coordinated water molecules and one lattice water molecule. The Co1 center is in an octahedral environment defined by two N atoms of one phen ligand, three O atoms of carboxylate groups from three BPTC ligands, and one O atom from one coordinated water molecule. The Co1–O bond lengths fall in the range 2.001 (3)–2.149 (3) Å and the two Co1-N distances are 2.108 (4) and 2.143 (4) Å, thus falling in the expected region (Konar, *et al.*,  2004). The Co2 atom is surrounded by six O atoms from four different BPTC ligands and one coordinated water molecule with Co–O distances in the range 2.025 (3)–2.210 (3) Å, and O–Co–O angles varying from 61.42 (11)°–168.23 (11)°. The octahedral coordination around the Co atoms is strongly distorted since the diametrical and non-diametrical bond angles indicate significant deviations from 180° and 90°, respectively. BPTC^4-^ forms eight coordination bonds with seven Co centers. Two carboxylates of BPTC^4-^ act as monodentate bridging and adopt a µ_2_-η^2^:η^0^ coordinated mode, one carboxylate acts as bidentate bridging and adopts a µ_2_-η^1^:η^1^ coordinated mode, while the remaining carboxylate chelates a Co cation. As a result, each BPTC^4-^ forms eight coordination bonds with seven Co centers, leading to a 2D layer structure parallel to the *ac* plane. Lattice water molecules occupy the space between 2D layers, and interact *via* interlayer O–H···O hydrogen bonds along the *b*-axis to generate a 3D supramolecular network (Table 1 and Fig.2).

### Experimental

A mixture of Co(NO_3_)_2_.6H_2_O (146 mg, 0.5 mmol),3,3',4,4' -biphenyltetracarboxylate (74 mg, 0.25 mmol), phen (99 mg, 0.5 mmol), NaOH (40 mg, 1.0 mmol) and water (15 ml) were heated at 393 K for 4 days in a sealed 25 ml Teflon-lined stainless steel vessel under autogenous pressure. Slow cooling of the reaction mixture at 2 K/min to room temperature gave salmon pink block crystals.

### Refinement

Hydrogen atoms attached to carbon were idealized and included as riding atoms; those attached to oxygen were located in the difference map, idealized and refined as riding. [*d*(O—H) = 0.85 Å; *U*_iso_(H) = 1.2*U*_eq_(O)]

### Crystal data

**Table:**

[Co_2_(C_16_H_6_O_8_)(C_12_H_8_N_2_)(H_2_O)_2_]·H_2_O	*Z* = 2
*M**_r_* = 678.32	*F*(000) = 688
Triclinic, *P*1	*D*_x_ = 1.770 Mg m^−^^3^
Hall symbol: -P 1	Mo *K*α radiation, λ = 0.71073 Å
*a* = 9.793 (3) Å	Cell parameters from 1160 reflections
*b* = 10.885 (3) Å	θ = 2.3–22.3°
*c* = 12.453 (3) Å	µ = 1.38 mm^−^^1^
α = 97.567 (4)°	*T* = 298 K
β = 102.608 (4)°	Block, red
γ = 95.653 (4)°	0.40 × 0.17 × 0.16 mm
*V* = 1273.1 (6) Å^3^	

### Data collection

**Table:**

Bruker SMART CCD area-detector diffractometer	4641 independent reflections
Radiation source: fine-focus sealed tube	3194 reflections with *I* > 2σ(*I*)
graphite	*R*_int_ = 0.034
φ and ω scans	θ_max_ = 25.5°, θ_min_ = 1.7°
Absorption correction: multi-scan (*SADABS*; Bruker, 2000)	*h* = −11→9
*T*_min_ = 0.609, *T*_max_ = 0.810	*k* = −9→13
6716 measured reflections	*l* = −14→15

### Refinement

**Table:**

Refinement on *F*^2^	Primary atom site location: structure-invariant direct methods
Least-squares matrix: full	Secondary atom site location: difference Fourier map
*R*[*F*^2^ > 2σ(*F*^2^)] = 0.052	Hydrogen site location: inferred from neighbouring sites
*wR*(*F*^2^) = 0.113	H-atom parameters constrained
*S* = 0.98	*w* = 1/[σ^2^(*F*_o_^2^) + (0.0467*P*)^2^] where *P* = (*F*_o_^2^ + 2*F*_c_^2^)/3
4641 reflections	(Δ/σ)_max_ = 0.001
388 parameters	Δρ_max_ = 0.46 e Å^−^^3^
0 restraints	Δρ_min_ = −0.50 e Å^−^^3^

### Special details

**Table:**

Geometry. All e.s.d.'s (except the e.s.d. in the dihedral angle between two l.s. planes) are estimated using the full covariance matrix. The cell e.s.d.'s are taken into account individually in the estimation of e.s.d.'s in distances, angles and torsion angles; correlations between e.s.d.'s in cell parameters are only used when they are defined by crystal symmetry. An approximate (isotropic) treatment of cell e.s.d.'s is used for estimating e.s.d.'s involving l.s. planes.
Refinement. Refinement of *F*^2^ against ALL reflections. The weighted *R*-factor *wR* and goodness of fit *S* are based on *F*^2^, conventional *R*-factors *R* are based on *F*, with *F* set to zero for negative *F*^2^. The threshold expression of *F*^2^ > σ(*F*^2^) is used only for calculating *R*-factors(gt) *etc*. and is not relevant to the choice of reflections for refinement. *R*-factors based on *F*^2^ are statistically about twice as large as those based on *F*, and *R*- factors based on ALL data will be even larger.

### Fractional atomic coordinates and isotropic or equivalent isotropic displacement parameters (Å^2^)

**Table:**

	*x*	*y*	*z*	*U*_iso_*/*U*_eq_	
C1	−0.1205 (5)	0.5902 (4)	0.6684 (4)	0.0227 (11)	
C2	−0.0391 (5)	0.6133 (4)	0.7872 (3)	0.0209 (10)	
C3	−0.0950 (5)	0.6775 (4)	0.8670 (4)	0.0284 (12)	
H3	−0.1796	0.7094	0.8450	0.034*	
C4	−0.0286 (5)	0.6957 (4)	0.9786 (4)	0.0298 (12)	
H4	−0.0699	0.7388	1.0300	0.036*	
C5	0.0979 (5)	0.6512 (4)	1.0160 (3)	0.0189 (10)	
C6	0.1527 (5)	0.5852 (4)	0.9355 (3)	0.0234 (11)	
H6	0.2372	0.5535	0.9583	0.028*	
C7	0.0872 (5)	0.5642 (4)	0.8227 (3)	0.0191 (10)	
C8	0.1543 (5)	0.4826 (5)	0.7467 (3)	0.0224 (11)	
C9	0.1711 (4)	0.6716 (4)	1.1364 (3)	0.0194 (10)	
C10	0.1249 (5)	0.7513 (4)	1.2133 (4)	0.0297 (12)	
H10	0.0491	0.7939	1.1891	0.036*	
C11	0.1904 (5)	0.7684 (5)	1.3263 (4)	0.0317 (12)	
H11	0.1585	0.8230	1.3765	0.038*	
C12	0.3014 (5)	0.7054 (4)	1.3644 (4)	0.0231 (11)	
C13	0.3634 (5)	0.7284 (5)	1.4884 (4)	0.0248 (11)	
C14	0.3493 (5)	0.6257 (4)	1.2883 (3)	0.0209 (10)	
C15	0.4728 (5)	0.5573 (4)	1.3218 (4)	0.0230 (11)	
C16	0.2826 (5)	0.6097 (4)	1.1761 (3)	0.0225 (10)	
H16	0.3144	0.5550	1.1260	0.027*	
C17	0.5542 (5)	0.7004 (5)	0.9733 (4)	0.0369 (13)	
H17	0.6056	0.6388	0.9503	0.044*	
C18	0.5861 (6)	0.7519 (5)	1.0863 (4)	0.0459 (15)	
H18	0.6582	0.7257	1.1365	0.055*	
C19	0.5106 (6)	0.8406 (5)	1.1219 (4)	0.0446 (15)	
H19	0.5308	0.8756	1.1968	0.054*	
C20	0.4023 (6)	0.8790 (5)	1.0452 (4)	0.0343 (13)	
C21	0.3795 (5)	0.8238 (4)	0.9332 (4)	0.0272 (11)	
C22	0.2701 (5)	0.8587 (4)	0.8500 (4)	0.0267 (11)	
C23	0.1811 (6)	0.9417 (5)	0.8828 (4)	0.0380 (13)	
C24	0.2065 (6)	0.9964 (5)	0.9968 (5)	0.0476 (15)	
H24	0.1481	1.0524	1.0184	0.057*	
C25	0.3128 (7)	0.9689 (5)	1.0736 (5)	0.0475 (15)	
H25	0.3290	1.0088	1.1469	0.057*	
C26	0.0685 (7)	0.9626 (5)	0.7995 (5)	0.0550 (17)	
H26	0.0046	1.0151	0.8173	0.066*	
C27	0.0522 (6)	0.9065 (6)	0.6930 (5)	0.0533 (17)	
H27	−0.0217	0.9215	0.6373	0.064*	
C28	0.1466 (6)	0.8265 (5)	0.6673 (4)	0.0381 (13)	
H28	0.1328	0.7869	0.5941	0.046*	
Co1	0.39769 (6)	0.67515 (6)	0.72127 (5)	0.02274 (18)	
Co2	0.24578 (6)	0.44660 (6)	0.52994 (5)	0.02263 (18)	
N1	0.2553 (4)	0.8043 (4)	0.7428 (3)	0.0277 (9)	
N2	0.4548 (4)	0.7350 (4)	0.8980 (3)	0.0273 (9)	
O1	−0.0796 (3)	0.5208 (3)	0.5955 (2)	0.0285 (8)	
O2	−0.2316 (3)	0.6395 (3)	0.6414 (2)	0.0304 (8)	
O3	0.1484 (4)	0.3712 (3)	0.7556 (3)	0.0385 (9)	
O4	0.2165 (3)	0.5344 (3)	0.6805 (2)	0.0229 (7)	
O5	0.4838 (3)	0.4631 (3)	1.2562 (2)	0.0284 (8)	
O6	0.5598 (3)	0.6015 (3)	1.4135 (2)	0.0264 (8)	
O7	0.4224 (4)	0.8357 (3)	1.5296 (2)	0.0352 (9)	
O8	0.3428 (3)	0.6420 (3)	1.5449 (2)	0.0217 (7)	
O9	0.1558 (4)	0.2681 (3)	0.5513 (2)	0.0376 (9)	
H9A	0.1290	0.2480	0.6078	0.045*	
H9B	0.1354	0.2042	0.5007	0.045*	
O10	0.5871 (3)	0.7894 (3)	0.7169 (2)	0.0306 (8)	
H10A	0.6525	0.7612	0.6901	0.037*	
H10B	0.5721	0.8574	0.6925	0.037*	
O11	0.3441 (5)	0.0884 (4)	0.5642 (4)	0.0968 (19)	
H11A	0.3480	0.0115	0.5697	0.116*	
H11B	0.4080	0.1111	0.5312	0.116*	

### Atomic displacement parameters (Å^2^)

**Table:**

	*U*^11^	*U*^22^	*U*^33^	*U*^12^	*U*^13^	*U*^23^
C1	0.015 (2)	0.028 (3)	0.023 (2)	−0.001 (2)	0.001 (2)	0.003 (2)
C2	0.019 (2)	0.027 (3)	0.014 (2)	0.003 (2)	−0.0016 (19)	0.003 (2)
C3	0.021 (3)	0.041 (3)	0.022 (3)	0.013 (2)	0.001 (2)	0.003 (2)
C4	0.033 (3)	0.038 (3)	0.018 (2)	0.011 (2)	0.006 (2)	−0.002 (2)
C5	0.021 (3)	0.021 (3)	0.015 (2)	0.004 (2)	0.0047 (19)	0.0034 (19)
C6	0.021 (3)	0.028 (3)	0.021 (2)	0.007 (2)	0.002 (2)	0.005 (2)
C7	0.023 (3)	0.025 (3)	0.011 (2)	0.004 (2)	0.0062 (18)	0.0035 (19)
C8	0.016 (2)	0.035 (3)	0.013 (2)	0.008 (2)	−0.0029 (19)	0.000 (2)
C9	0.019 (2)	0.024 (3)	0.015 (2)	0.004 (2)	0.0021 (19)	0.003 (2)
C10	0.034 (3)	0.032 (3)	0.023 (3)	0.017 (2)	0.001 (2)	0.005 (2)
C11	0.040 (3)	0.040 (3)	0.016 (2)	0.022 (3)	0.004 (2)	−0.001 (2)
C12	0.024 (3)	0.022 (3)	0.023 (2)	0.001 (2)	0.004 (2)	0.006 (2)
C13	0.025 (3)	0.034 (3)	0.016 (2)	0.016 (2)	0.001 (2)	0.001 (2)
C14	0.023 (3)	0.025 (3)	0.015 (2)	0.007 (2)	0.0029 (19)	0.005 (2)
C15	0.016 (2)	0.034 (3)	0.022 (2)	0.007 (2)	0.008 (2)	0.010 (2)
C16	0.024 (3)	0.024 (3)	0.019 (2)	0.006 (2)	0.002 (2)	0.002 (2)
C17	0.033 (3)	0.050 (4)	0.026 (3)	0.015 (3)	0.002 (2)	0.004 (3)
C18	0.045 (4)	0.062 (4)	0.024 (3)	0.004 (3)	−0.004 (3)	0.005 (3)
C19	0.055 (4)	0.049 (4)	0.022 (3)	−0.014 (3)	0.007 (3)	−0.003 (3)
C20	0.042 (3)	0.036 (3)	0.023 (3)	−0.006 (3)	0.012 (2)	−0.004 (2)
C21	0.032 (3)	0.027 (3)	0.021 (2)	−0.004 (2)	0.009 (2)	−0.002 (2)
C22	0.031 (3)	0.021 (3)	0.028 (3)	0.003 (2)	0.008 (2)	0.001 (2)
C23	0.052 (4)	0.028 (3)	0.039 (3)	0.012 (3)	0.017 (3)	0.004 (2)
C24	0.066 (4)	0.035 (3)	0.050 (4)	0.017 (3)	0.033 (3)	−0.006 (3)
C25	0.065 (4)	0.039 (4)	0.036 (3)	0.001 (3)	0.019 (3)	−0.012 (3)
C26	0.068 (5)	0.045 (4)	0.063 (4)	0.034 (3)	0.027 (4)	0.012 (3)
C27	0.056 (4)	0.064 (4)	0.047 (4)	0.035 (3)	0.011 (3)	0.017 (3)
C28	0.045 (4)	0.043 (4)	0.028 (3)	0.015 (3)	0.010 (3)	0.004 (3)
Co1	0.0226 (4)	0.0293 (4)	0.0144 (3)	0.0090 (3)	0.0007 (3)	−0.0016 (3)
Co2	0.0197 (4)	0.0316 (4)	0.0136 (3)	0.0072 (3)	−0.0016 (3)	−0.0011 (3)
N1	0.032 (2)	0.030 (2)	0.023 (2)	0.0107 (19)	0.0061 (18)	0.0034 (18)
N2	0.024 (2)	0.035 (3)	0.022 (2)	0.0073 (19)	0.0032 (18)	0.0003 (19)
O1	0.0256 (19)	0.041 (2)	0.0142 (16)	0.0109 (16)	−0.0018 (14)	−0.0061 (15)
O2	0.0232 (19)	0.050 (2)	0.0155 (16)	0.0147 (16)	−0.0021 (14)	0.0003 (15)
O3	0.057 (3)	0.033 (2)	0.032 (2)	0.0186 (19)	0.0175 (18)	0.0101 (17)
O4	0.0182 (17)	0.033 (2)	0.0159 (15)	0.0016 (14)	0.0036 (13)	−0.0008 (14)
O5	0.030 (2)	0.033 (2)	0.0183 (16)	0.0156 (16)	−0.0006 (14)	−0.0055 (15)
O6	0.0196 (18)	0.039 (2)	0.0164 (16)	0.0134 (15)	−0.0035 (14)	−0.0042 (15)
O7	0.047 (2)	0.024 (2)	0.0256 (18)	−0.0012 (17)	−0.0046 (16)	−0.0016 (16)
O8	0.0254 (18)	0.0271 (19)	0.0116 (15)	0.0054 (14)	0.0011 (13)	0.0031 (14)
O9	0.052 (2)	0.033 (2)	0.0264 (18)	−0.0014 (17)	0.0126 (17)	−0.0021 (16)
O10	0.031 (2)	0.030 (2)	0.0276 (18)	0.0052 (15)	0.0039 (15)	−0.0027 (15)
O11	0.104 (4)	0.038 (3)	0.184 (5)	0.023 (3)	0.094 (4)	0.031 (3)

### Geometric parameters (Å, °)

**Table:**

C1—O2	1.260 (5)	C19—H19	0.9300
C1—O1	1.262 (5)	C20—C21	1.404 (6)
C1—C2	1.495 (6)	C20—C25	1.437 (7)
C1—Co2^i^	2.467 (4)	C21—N2	1.357 (6)
C2—C3	1.381 (6)	C21—C22	1.437 (6)
C2—C7	1.402 (6)	C22—N1	1.358 (5)
C3—C4	1.379 (6)	C22—C23	1.399 (7)
C3—H3	0.9300	C23—C26	1.398 (7)
C4—C5	1.383 (6)	C23—C24	1.424 (7)
C4—H4	0.9300	C24—C25	1.337 (8)
C5—C6	1.389 (6)	C24—H24	0.9300
C5—C9	1.491 (5)	C25—H25	0.9300
C6—C7	1.390 (6)	C26—C27	1.354 (7)
C6—H6	0.9300	C26—H26	0.9300
C7—C8	1.509 (6)	C27—C28	1.389 (7)
C8—O3	1.229 (5)	C27—H27	0.9300
C8—O4	1.283 (5)	C28—N1	1.322 (6)
C9—C16	1.374 (6)	C28—H28	0.9300
C9—C10	1.386 (6)	Co1—O5^ii^	2.001 (3)
C10—C11	1.393 (6)	Co1—N1	2.108 (4)
C10—H10	0.9300	Co1—O8^iii^	2.117 (3)
C11—C12	1.373 (6)	Co1—N2	2.143 (4)
C11—H11	0.9300	Co1—O10	2.144 (3)
C12—C14	1.388 (6)	Co1—O4	2.149 (3)
C12—C13	1.508 (6)	Co2—O6^ii^	2.025 (3)
C13—O7	1.241 (5)	Co2—O4	2.080 (3)
C13—O8	1.273 (5)	Co2—O1^i^	2.088 (3)
C14—C16	1.387 (6)	Co2—O9	2.127 (3)
C14—C15	1.493 (6)	Co2—O2^i^	2.184 (3)
C15—O5	1.253 (5)	Co2—O8^iii^	2.210 (3)
C15—O6	1.269 (5)	Co2—C1^i^	2.467 (4)
C16—H16	0.9300	O9—H9A	0.8500
C17—N2	1.318 (6)	O9—H9B	0.8499
C17—C18	1.399 (6)	O10—H10A	0.8499
C17—H17	0.9300	O10—H10B	0.8501
C18—C19	1.359 (8)	O11—H11A	0.8520
C18—H18	0.9300	O11—H11B	0.8548
C19—C20	1.402 (7)		
			
O2—C1—O1	119.9 (4)	C25—C24—H24	119.3
O2—C1—C2	119.5 (4)	C23—C24—H24	119.3
O1—C1—C2	120.6 (4)	C24—C25—C20	121.3 (5)
O2—C1—Co2^i^	62.1 (2)	C24—C25—H25	119.3
O1—C1—Co2^i^	57.8 (2)	C20—C25—H25	119.3
C2—C1—Co2^i^	177.7 (3)	C27—C26—C23	120.1 (5)
C3—C2—C7	118.1 (4)	C27—C26—H26	119.9
C3—C2—C1	119.1 (4)	C23—C26—H26	119.9
C7—C2—C1	122.6 (4)	C26—C27—C28	119.6 (5)
C4—C3—C2	121.7 (4)	C26—C27—H27	120.2
C4—C3—H3	119.1	C28—C27—H27	120.2
C2—C3—H3	119.1	N1—C28—C27	122.6 (5)
C3—C4—C5	121.6 (4)	N1—C28—H28	118.7
C3—C4—H4	119.2	C27—C28—H28	118.7
C5—C4—H4	119.2	O5^ii^—Co1—N1	161.87 (14)
C4—C5—C6	116.5 (4)	O5^ii^—Co1—O8^iii^	97.71 (11)
C4—C5—C9	121.9 (4)	N1—Co1—O8^iii^	96.93 (13)
C6—C5—C9	121.6 (4)	O5^ii^—Co1—N2	88.47 (13)
C5—C6—C7	123.2 (4)	N1—Co1—N2	77.93 (14)
C5—C6—H6	118.4	O8^iii^—Co1—N2	172.24 (13)
C7—C6—H6	118.4	O5^ii^—Co1—O10	86.77 (13)
C6—C7—C2	118.9 (4)	N1—Co1—O10	104.03 (14)
C6—C7—C8	116.8 (4)	O8^iii^—Co1—O10	89.41 (11)
C2—C7—C8	124.2 (4)	N2—Co1—O10	86.25 (13)
O3—C8—O4	124.7 (4)	O5^ii^—Co1—O4	87.22 (13)
O3—C8—C7	117.0 (4)	N1—Co1—O4	86.00 (14)
O4—C8—C7	118.2 (4)	O8^iii^—Co1—O4	76.00 (11)
C16—C9—C10	117.5 (4)	N2—Co1—O4	109.13 (13)
C16—C9—C5	121.9 (4)	O10—Co1—O4	163.32 (11)
C10—C9—C5	120.6 (4)	O6^ii^—Co2—O4	98.38 (11)
C9—C10—C11	121.0 (4)	O6^ii^—Co2—O1^i^	153.17 (12)
C9—C10—H10	119.5	O4—Co2—O1^i^	107.16 (12)
C11—C10—H10	119.5	O6^ii^—Co2—O9	89.10 (13)
C12—C11—C10	120.7 (4)	O4—Co2—O9	93.31 (12)
C12—C11—H11	119.7	O1^i^—Co2—O9	97.35 (13)
C10—C11—H11	119.7	O6^ii^—Co2—O2^i^	93.38 (11)
C11—C12—C14	119.0 (4)	O4—Co2—O2^i^	168.19 (12)
C11—C12—C13	116.7 (4)	O1^i^—Co2—O2^i^	61.42 (11)
C14—C12—C13	124.3 (4)	O9—Co2—O2^i^	85.65 (12)
O7—C13—O8	124.2 (4)	O6^ii^—Co2—O8^iii^	89.12 (12)
O7—C13—C12	116.5 (4)	O4—Co2—O8^iii^	75.45 (11)
O8—C13—C12	119.0 (4)	O1^i^—Co2—O8^iii^	89.44 (12)
C16—C14—C12	119.4 (4)	O9—Co2—O8^iii^	168.23 (11)
C16—C14—C15	117.9 (4)	O2^i^—Co2—O8^iii^	106.07 (12)
C12—C14—C15	122.6 (4)	O6^ii^—Co2—C1^i^	123.70 (13)
O5—C15—O6	125.4 (4)	O4—Co2—C1^i^	137.77 (14)
O5—C15—C14	117.5 (4)	O1^i^—Co2—C1^i^	30.76 (13)
O6—C15—C14	117.0 (4)	O9—Co2—C1^i^	90.96 (13)
C9—C16—C14	122.4 (4)	O2^i^—Co2—C1^i^	30.68 (13)
C9—C16—H16	118.8	O8^iii^—Co2—C1^i^	99.70 (13)
C14—C16—H16	118.8	C28—N1—C22	117.8 (4)
N2—C17—C18	123.2 (5)	C28—N1—Co1	127.4 (3)
N2—C17—H17	118.4	C22—N1—Co1	114.3 (3)
C18—C17—H17	118.4	C17—N2—C21	117.7 (4)
C19—C18—C17	119.3 (5)	C17—N2—Co1	128.8 (3)
C19—C18—H18	120.4	C21—N2—Co1	113.5 (3)
C17—C18—H18	120.4	C1—O1—Co2^i^	91.4 (3)
C18—C19—C20	119.6 (5)	C1—O2—Co2^i^	87.2 (3)
C18—C19—H19	120.2	C8—O4—Co2	125.8 (3)
C20—C19—H19	120.2	C8—O4—Co1	128.5 (3)
C19—C20—C21	117.1 (5)	Co2—O4—Co1	98.35 (12)
C19—C20—C25	124.6 (5)	C15—O5—Co1^ii^	131.1 (3)
C21—C20—C25	118.3 (5)	C15—O6—Co2^ii^	127.3 (3)
N2—C21—C20	123.1 (5)	C13—O8—Co1^iv^	121.5 (3)
N2—C21—C22	116.8 (4)	C13—O8—Co2^iv^	142.9 (3)
C20—C21—C22	120.1 (5)	Co1^iv^—O8—Co2^iv^	95.37 (11)
N1—C22—C23	123.3 (5)	Co2—O9—H9A	128.0
N1—C22—C21	117.4 (4)	Co2—O9—H9B	124.2
C23—C22—C21	119.3 (4)	H9A—O9—H9B	107.7
C26—C23—C22	116.5 (5)	Co1—O10—H10A	123.2
C26—C23—C24	124.1 (5)	Co1—O10—H10B	113.4
C22—C23—C24	119.4 (5)	H10A—O10—H10B	107.7
C25—C24—C23	121.4 (5)	H11A—O11—H11B	107.1
			
O2—C1—C2—C3	6.6 (7)	C26—C27—C28—N1	−1.6 (9)
O1—C1—C2—C3	−172.6 (4)	C27—C28—N1—C22	2.8 (8)
O2—C1—C2—C7	−178.3 (4)	C27—C28—N1—Co1	174.4 (4)
O1—C1—C2—C7	2.5 (7)	C23—C22—N1—C28	−3.7 (7)
C7—C2—C3—C4	1.1 (7)	C21—C22—N1—C28	173.6 (4)
C1—C2—C3—C4	176.4 (4)	C23—C22—N1—Co1	−176.4 (4)
C2—C3—C4—C5	0.5 (8)	C21—C22—N1—Co1	0.9 (5)
C3—C4—C5—C6	−1.4 (7)	O5^ii^—Co1—N1—C28	−129.0 (5)
C3—C4—C5—C9	179.3 (4)	O8^iii^—Co1—N1—C28	14.7 (4)
C4—C5—C6—C7	0.6 (7)	N2—Co1—N1—C28	−171.2 (5)
C9—C5—C6—C7	179.9 (4)	O10—Co1—N1—C28	105.8 (4)
C5—C6—C7—C2	1.0 (7)	O4—Co1—N1—C28	−60.7 (4)
C5—C6—C7—C8	−175.6 (4)	O5^ii^—Co1—N1—C22	42.8 (6)
C3—C2—C7—C6	−1.9 (7)	O8^iii^—Co1—N1—C22	−173.5 (3)
C1—C2—C7—C6	−176.9 (4)	N2—Co1—N1—C22	0.6 (3)
C3—C2—C7—C8	174.5 (4)	O10—Co1—N1—C22	−82.4 (3)
C1—C2—C7—C8	−0.6 (7)	O4—Co1—N1—C22	111.2 (3)
C6—C7—C8—O3	68.8 (6)	C18—C17—N2—C21	−0.8 (7)
C2—C7—C8—O3	−107.7 (5)	C18—C17—N2—Co1	176.4 (4)
C6—C7—C8—O4	−109.2 (5)	C20—C21—N2—C17	−0.4 (7)
C2—C7—C8—O4	74.4 (6)	C22—C21—N2—C17	−179.0 (4)
C4—C5—C9—C16	168.3 (5)	C20—C21—N2—Co1	−178.1 (4)
C6—C5—C9—C16	−10.9 (7)	C22—C21—N2—Co1	3.3 (5)
C4—C5—C9—C10	−9.3 (7)	O5^ii^—Co1—N2—C17	12.6 (4)
C6—C5—C9—C10	171.5 (4)	N1—Co1—N2—C17	−179.5 (5)
C16—C9—C10—C11	0.6 (7)	O10—Co1—N2—C17	−74.3 (4)
C5—C9—C10—C11	178.3 (4)	O4—Co1—N2—C17	99.1 (4)
C9—C10—C11—C12	−0.8 (8)	O5^ii^—Co1—N2—C21	−170.1 (3)
C10—C11—C12—C14	1.1 (7)	N1—Co1—N2—C21	−2.1 (3)
C10—C11—C12—C13	−178.9 (4)	O10—Co1—N2—C21	103.1 (3)
C11—C12—C13—O7	−65.5 (6)	O4—Co1—N2—C21	−83.5 (3)
C14—C12—C13—O7	114.5 (5)	O2—C1—O1—Co2^i^	2.7 (4)
C11—C12—C13—O8	108.9 (5)	C2—C1—O1—Co2^i^	−178.1 (4)
C14—C12—C13—O8	−71.1 (6)	O1—C1—O2—Co2^i^	−2.6 (4)
C11—C12—C14—C16	−1.2 (7)	C2—C1—O2—Co2^i^	178.2 (4)
C13—C12—C14—C16	178.8 (4)	O3—C8—O4—Co2	28.2 (6)
C11—C12—C14—C15	177.6 (4)	C7—C8—O4—Co2	−154.1 (3)
C13—C12—C14—C15	−2.4 (7)	O3—C8—O4—Co1	−115.2 (4)
C16—C14—C15—O5	−21.1 (6)	C7—C8—O4—Co1	62.6 (5)
C12—C14—C15—O5	160.0 (4)	O6^ii^—Co2—O4—C8	−93.6 (3)
C16—C14—C15—O6	157.3 (4)	O1^i^—Co2—O4—C8	94.8 (3)
C12—C14—C15—O6	−21.6 (7)	O9—Co2—O4—C8	−4.0 (3)
C10—C9—C16—C14	−0.8 (7)	O2^i^—Co2—O4—C8	80.6 (7)
C5—C9—C16—C14	−178.5 (4)	O8^iii^—Co2—O4—C8	179.6 (3)
C12—C14—C16—C9	1.1 (7)	C1^i^—Co2—O4—C8	91.1 (4)
C15—C14—C16—C9	−177.8 (4)	O6^ii^—Co2—O4—Co1	58.29 (14)
N2—C17—C18—C19	1.0 (8)	O1^i^—Co2—O4—Co1	−113.39 (13)
C17—C18—C19—C20	0.0 (8)	O9—Co2—O4—Co1	147.87 (13)
C18—C19—C20—C21	−1.1 (8)	O2^i^—Co2—O4—Co1	−127.6 (6)
C18—C19—C20—C25	177.9 (5)	O8^iii^—Co2—O4—Co1	−28.59 (11)
C19—C20—C21—N2	1.4 (7)	C1^i^—Co2—O4—Co1	−117.05 (18)
C25—C20—C21—N2	−177.8 (5)	O5^ii^—Co1—O4—C8	82.0 (4)
C19—C20—C21—C22	179.9 (4)	N1—Co1—O4—C8	−81.2 (4)
C25—C20—C21—C22	0.8 (7)	O8^iii^—Co1—O4—C8	−179.4 (4)
N2—C21—C22—N1	−2.9 (6)	N2—Co1—O4—C8	−5.5 (4)
C20—C21—C22—N1	178.4 (4)	O10—Co1—O4—C8	150.9 (4)
N2—C21—C22—C23	174.5 (5)	O5^ii^—Co1—O4—Co2	−68.77 (12)
C20—C21—C22—C23	−4.1 (7)	N1—Co1—O4—Co2	128.06 (14)
N1—C22—C23—C26	3.2 (8)	O8^iii^—Co1—O4—Co2	29.89 (11)
C21—C22—C23—C26	−174.1 (5)	N2—Co1—O4—Co2	−156.19 (13)
N1—C22—C23—C24	−178.6 (5)	O10—Co1—O4—Co2	0.2 (5)
C21—C22—C23—C24	4.2 (8)	O6—C15—O5—Co1^ii^	16.8 (7)
C26—C23—C24—C25	177.3 (6)	C14—C15—O5—Co1^ii^	−165.0 (3)
C22—C23—C24—C25	−0.8 (9)	O5—C15—O6—Co2^ii^	10.6 (7)
C23—C24—C25—C20	−2.6 (9)	C14—C15—O6—Co2^ii^	−167.6 (3)
C19—C20—C25—C24	−176.4 (5)	O7—C13—O8—Co1^iv^	3.7 (6)
C21—C20—C25—C24	2.6 (8)	C12—C13—O8—Co1^iv^	−170.3 (3)
C22—C23—C26—C27	−1.8 (9)	O7—C13—O8—Co2^iv^	176.9 (3)
C24—C23—C26—C27	−180.0 (6)	C12—C13—O8—Co2^iv^	3.0 (7)
C23—C26—C27—C28	1.1 (9)		

Symmetry codes: (i) −*x*, −*y*+1, −*z*+1; (ii) −*x*+1, −*y*+1, −*z*+2; (iii) *x*, *y*, *z*−1; (iv) *x*, *y*, *z*+1.

### Hydrogen-bond geometry (Å, °)

**Table:**

*D*—H···*A*	*D*—H	H···*A*	*D*···*A*	*D*—H···*A*
O11—H11B···O7^ii^	0.85	2.04	2.886 (6)	173.
O11—H11A···O7^v^	0.85	2.15	2.931 (5)	152.
O10—H10B···O7^iii^	0.85	2.20	2.668 (4)	115.
O10—H10A···O2^vi^	0.85	1.95	2.756 (4)	159.
O9—H9A···O3	0.85	2.09	2.661 (4)	124.

Symmetry codes: (ii) −*x*+1, −*y*+1, −*z*+2; (v) *x*, *y*−1, *z*−1; (iii) *x*, *y*, *z*−1; (vi) *x*+1, *y*, *z*.

## References

[bb1] Bruker (2000). *SMART*, *SAINT* and *SADABS* Bruker AXS Inc., Madison, Wisconsin, USA.

[bb2] Konar, S., Zangrando, E., Drew, M. G. B., Ribas, J. & Chaudhuri, N. R. (2004). *Dalton Trans* pp. 260–266. 10.1039/b311988b 15356721

[bb3] Rowsell, J. L. C. & Yaghi, O. M. (2005). *Angew. Chem. Int. Ed.* **44**, 4670–4679. 10.1002/anie.200462786 16028207

[bb4] Sheldrick, G. M. (2008). *Acta Cryst.* A**64**, 112–122. 10.1107/S0108767307043930 18156677

[bb5] Zhu, S. R., Zhang, H., Shao, M., Zhao, Y. M. & Li, M. X. (2008). *Transition Met. Chem* **33**, 669–680.

